# Choosing Wisely Campaign – Innovations in Cardiovascular Science and The United States Healthcare System

**DOI:** 10.7759/cureus.2931

**Published:** 2018-07-05

**Authors:** Hiba Rehman, Fahad Ali, Muhammad Asif Mangi

**Affiliations:** 1 Internal Medicine, Hamdard University Hospital, Karachi, PAK; 2 Cardiology, Lehigh Valley Hospital Network, Allentown, USA; 3 Internal Medicine, Orange Park Medical Center, Orange Park, USA

**Keywords:** united states healthcare, economic impacts, choosing wisely campaign

## Abstract

The United States (US) is the third most expensive health care system in the world, but despite that, the US ranked last in the top 50 countries of the world when it comes to the performance measures, such as healthcare efficiency, life expectancy, health care costs, and gross domestic product (GDP) percentage. The spending health care cost keeps increasing and most of the healthcare costs go to waste. Due to this reason, it is therefore extremely important to focus on improving the quality and to bring the costs in appropriate control. To avoid this issue, the Choosing Wisely Campaign (CWC) came into being in 2012. The CWC encourages discussions between providers and patients regarding the care based on the evidence base, free from harm, duplicative or redundant tests/procedures that the patient already received, and whether medications, tests, or procedures are really necessary. Although diagnostic tests or procedures are highly valued for decision-making, unnecessary testing creates harmful health services and an economic impact on the healthcare system. The CWC has spread widely throughout the world but has many challenges which are limiting the CWC in further adoption and spread in the US. To overcome challenges in implementing and spreading the CWC, the government, physicians, social media, and mass media play an important role.

## Introduction and background

According to an estimate, about 20% - 30% of all healthcare spending is attributed to waste in health care [1-3]. Decisions made by health care providers (including those made with patients’ consent) contribute to about 80% of healthcare costs [4]. This means that providers contribute to a significant proportion of wasted health care dollars. This waste may be in the form of unnecessary radiological or laboratory investigation or new expensive medication or overzealous investigative approach out of fear of legal action or lawsuits. With the growing healthcare costs in the US, the national economy is under more strain. For this reason, serious actions need to be taken. Various associations and bodies have put forth several suggestions. Some suggest that the high cost of health care is due to an inappropriate legal system and not entirely due to the medical system; based on this premise, the idea of legal reforms was suggested [5-6]. Some people gave a slightly different view. In late 2009, Dr. Brody put forth the idea of “top 5 list” in his paper published by the New England Journal of Medicine. He proposed that each major medical society needs to come up with a list of tests, procedures, or treatments which are expensive and commonly ordered by the providers but have not been shown by scientific evidence to have any significant benefit at least in the common scenarios, if not in exceptional cases [6]. He further suggested that once the “Top 5 list” is agreed upon, then each specialty society needs to provide guidelines or action plans to educate the medical community to discourage the use of things in the “top 5 list” in the specific patient population. In this way, not only a significant revenue of health care could be saved without compromising patient care, but also patients may be saved from going through unnecessary procedures or investigations. Dr. Brody’s opinions were taken very well by the professional community. The US National Physicians Alliance (NPA), with the assistance of the American Board of Internal Medicine (ABIM), launched a project titled “Promoting Good Stewardship in Clinical Practice”. In 2011, this project designed a “top 5 list” for each specialty of internal medicine, family practice, and pediatrics [7]. The ABIM Foundation supported this project through the “Putting The Charter Into Practice Grant” [8]. The Consumer Reports group also collaborated on this project, along with specialty societies and the ABIM [2]. Since the launch of the Choosing Wisely Campaign (CWC) in 2012, it has spread to more than 20 countries worldwide. The number of specialty societies participating in this campaign has increased, and to date, there are more than 540 specialty society recommendations and 150 patient resources now [9]. Though there are a remarkable number of specialties recommendations, the CWC faces many challenges and barriers in implementing and spreading this campaign in the US. In this article, we will be focusing on the aims of CWC, Choosing Wisely (CW) and cardiovascular science, the economic impact of the US healthcare system, and barriers in adopting and spreading CW.

## Review

Aims of the Choosing Wisely Campaign

The aim of the CWC is to encourage discussions between providers and consumers regarding the care that a patient is going to get is (a) based on evidence, (b) free from harm, (c) duplicative or redundant test/procedure that the patient already received, and (d) whether it is really necessary. 

Another aim of this initiative is to reach out to the medical community and healthcare providers, as well the consumers or patients, and to educate them about these lists and to initiate a discussion between patients and their healthcare providers before going through the procedures mentioned in these lists. It is also important to understand that these recommendations are general guidelines and the providers need to consider them depending on individual scenarios and patient discussion. On the other hand, Consumer Reports and consumer groups are developing materials to better inform the patients so that patients can discuss these matters in better detail with their providers. Considering the fact that every patient situation is unique and treatment plans may vary depending on the situation, it is therefore emphasized that these lists need not be considered as absolute exclusion or inclusion criteria. Instead, a general overview may be drawn from these lists, and under the light of these guidelines, patients need to be assessed on an individual basis for an appropriate management plan [9].

The CWC has begun to change physician attitude to avoid overuse of health resources. The physician often uses modern medicine to help the patient which, in turn, leads to higher costs when it is not necessary. The adoption of this campaign requires more evidence of effective outcomes.

Choosing Wisely cardiovascular science

According to Consumer Reports, 44% of healthy adults have unnecessary heart screening tests [10]. A survey reported a large number of consumers were undergoing wasteful heart screening investigations without any effective reason from a provider for the test [10]. In this survey, they included people with no history of hypertension, hyperlipidemia, heart disease, and smoking. In fact, they were labeled in good to excellent health condition. Of that group, 39% had an electrocardiogram (EKG), 10% had echocardiography (echo), and 12% had a stress test. Surprisingly, only 17% knew the test reason, and 11% knew what could be done if it were abnormal. In 2016, a systemic review was published in the Journal of the American Medical Association (JAMA) “2016 Update on Medical Overuse”, which further stratified medical provider waste into three different groups, including the overuse of testing, overtreatment, and medical practices to question [11]. Normally, guidelines do not recommend hospitalization for low-risk syncope [12]. However, 34% of admissions were low-risk syncope per this review, out of which 88% had a head computed tomography (CT) scan, 19% had magnetic resonance imaging (MRI), 64% had an echo, and 93% had telemetry monitoring. Similarly, over-hospitalization and overtreatment for syncope were found in a survey in which 83% of patients had unnecessary testing [13].

Unnecessary anticoagulation was also commonly seen in young and healthy patients with nonvalvular atrial fibrillation [14]. The National Cardiovascular Data Registry Practice Innovation and Clinical Excellence Registry of over 10,000 patients with nonvalvular atrial fibrillation were shown to have had unnecessary anticoagulation. In this registry, patients with a CHADS_2_ score of 0 (23.3%) and CHA_2_DS_2_-VASc score 0 (26.6%) were prescribed anticoagulation. Guidelines recommend avoiding anticoagulation in a patient with a CHADS_2_ score and CHA_2_DS_2_VASc score of 0.

Further, Dr. Hughes discovered that one-third of the outpatient stress test was not recommended as they were considered low-risk [15]. Therefore, Hughes et al. developed a tool, “The Guide to Ordering Stress Tests for Suspected Coronary Artery Disease”, to help to identify the need for a stress test for the referring physician. This tool has worked extremely well and outpatient stress tests have dropped from 33% to 5%.

Barriers to Choosing Wisely

There are many barriers to the CWC, but the main barrier is the physician awareness about choosing wisely. Colla, et al. in his survey of “Physician Perceptions of Choosing Wisely and Drivers of Overuse” found that primary care physicians reported significantly greater awareness of Choosing Wisely (47.2%) than medical specialists (37.4%) and surgical specialists (27%) [16]. In addition to awareness, another common barrier is defensive medicine [17-18]. The Massachusetts general survey of various specialty found that there was 21% - 31% of defensive medicine in various ways [18]. Rothenberg et al. further stratify the same idea of defensive medicine and found 28% of the orders and 13% of the costs were partially defensive and almost 3% completely defensive [17]. However, this study found a lower percentage of defensive medicine, but this study had some limitations, including lack of anonymity, small sample size, the inclusion of only hospital medicine service at three hospitals in the health system, and its subjective nature. To conclude, although a higher proportion of hospital orders had some defensive component, this study found that there were some orders which were absolutely defensive and physicians behavior about defensive medicine did not correlate with the costs.

There was another study published in JAMA “Views of US physicians about controlling health care cost”. The idea of this study was to assess physicians' attitude toward and perceived role in addressing health care costs [19]. Many of the physicians believe that health insurance companies (56%), pharmaceutical and device manufacturers (56%), trial lawyers (60%), hospitals and health systems (56%), and patients (52%) have a “major responsibility” for reducing costs, whereas only 36% reported that practicing physicians have a ”major responsibility”. Other barriers to choosing wisely are included in Table 1 [20-21].

**Table 1:** Common Barriers to Choosing Wisely

Barriers to Choosing Wisely
Medical malpractice
Patient requests for tests and treatment
Physician recommends unnecessary tests and treatment
Lack of physician time for shared decision making
Lack of time to assess patient benefit from service
Reward with ordering more services (Performance measure)
Reward ordering more services (Payment policies)
Lack of automated decision support to assess patient benefit from service
Cost of medications
Patient preference of brand name over generic medication
Insurance Issues

Economic impact

The escalating health care costs are a significant problem in developed nations. Between 2000 and 2010, the average healthcare expenditure per capita increased by more than 70% in about 34 developed nations. US healthcare expenditure reached about $3 trillion in 2014 from $2.59 trillion in 2010 and $1.37 trillion in 2000, about 17.5% of the nation’s gross domestic product (GDP) in 2014 [22], which has further increased to $3.6 trillion with 18% of nation’s GDP in February 2018 [23]. Approximately 32% of this health care expenditure is consumed in hospital care. This expenditure on hospital care is influenced by prices and also by the use and intensity of services [24].

The US has stronger economic growth, but limited success in controlling health care expenditures. There are multiple reasons for this cost pressure, including new and expensive drugs, novel medical devices, procedures to aid with chronic conditions, growing long-term needs, higher wages for a provider with a shortage of provider, and higher costs from insurance companies [25].

Although a drop in health care costs was seen in 2013, this drop is mainly attributed to the recent global financial crises. Some credit also goes to the Affordable Care Act (ACA) [26] as there were cuts in Medicare payments to health care providers and insurers, which helped to limit the spending of health care in 2013. The main motivation behind the ACA was to control spending costs. Nevertheless, a return to the previous state of increasing health care spending growth is imminent, as suggested by the experts. Health care costs are growing slowly; it has now reached 4.9% in February 2018 compared to 2.1% in March 2013 [23]. If a higher state is reached like that seen in 2015, then it will lead to further stress on the national economy. Therefore, it is very important to take action to bring health care costs in control as it is already putting an increased strain on the economy [27]. According to a 2015 Bloomberg report, the US healthcare system is third most expensive health care system in the world since the per capita spending in the US is higher than any other country, only surpassed by Norway and Switzerland [28]. However, when the report ranked 55 developed nations in the world in terms of life expectancy, health care efficiency, healthcare cost per capita, and a GDP percentage, the US healthcare system ranked near the bottom at 50.

**Figure 1:**
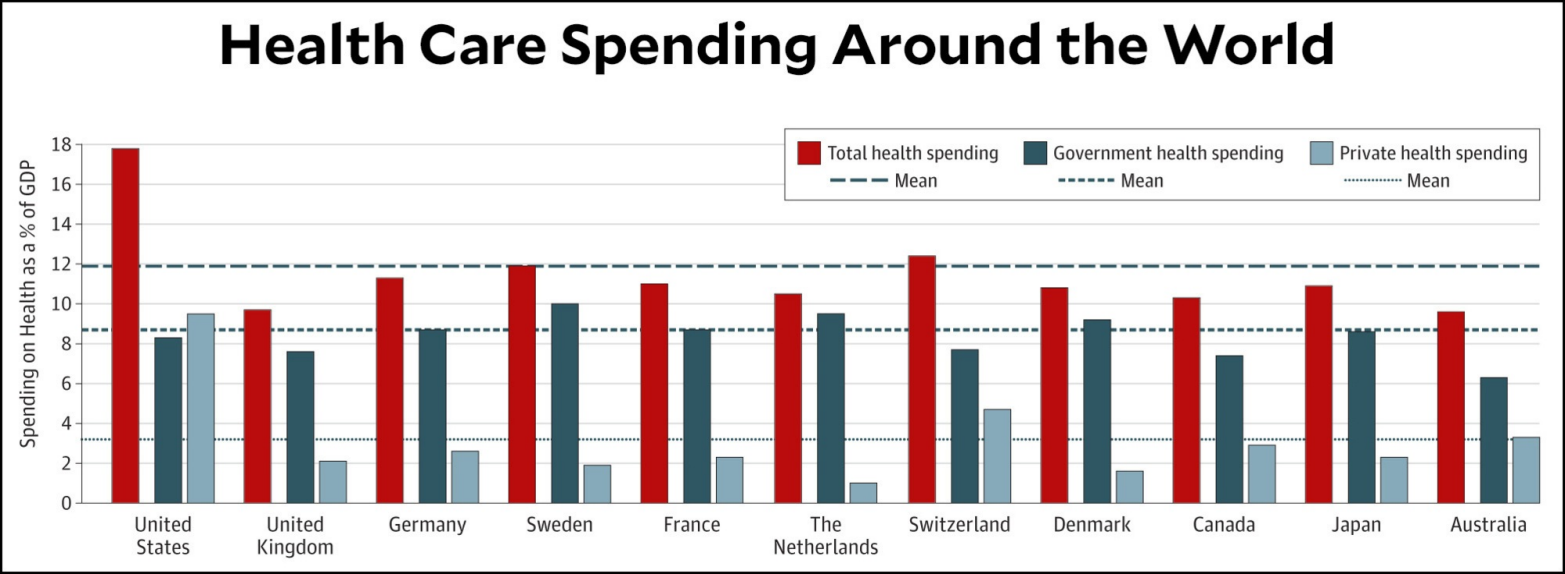
Health Care Spending Around the World Adopted from Reference #29

Further, in comparison to other countries, US consumers pay more for provider services, hospital services, drugs, and diagnostic tests [29]. In the US, the inpatient medication price is two times more than in Canada; MRIs cost three times more than in Australia, and the price for coronary artery bypass is four times more expensive than in the Netherlands (Figure 1) [30].

**Figure 2:**
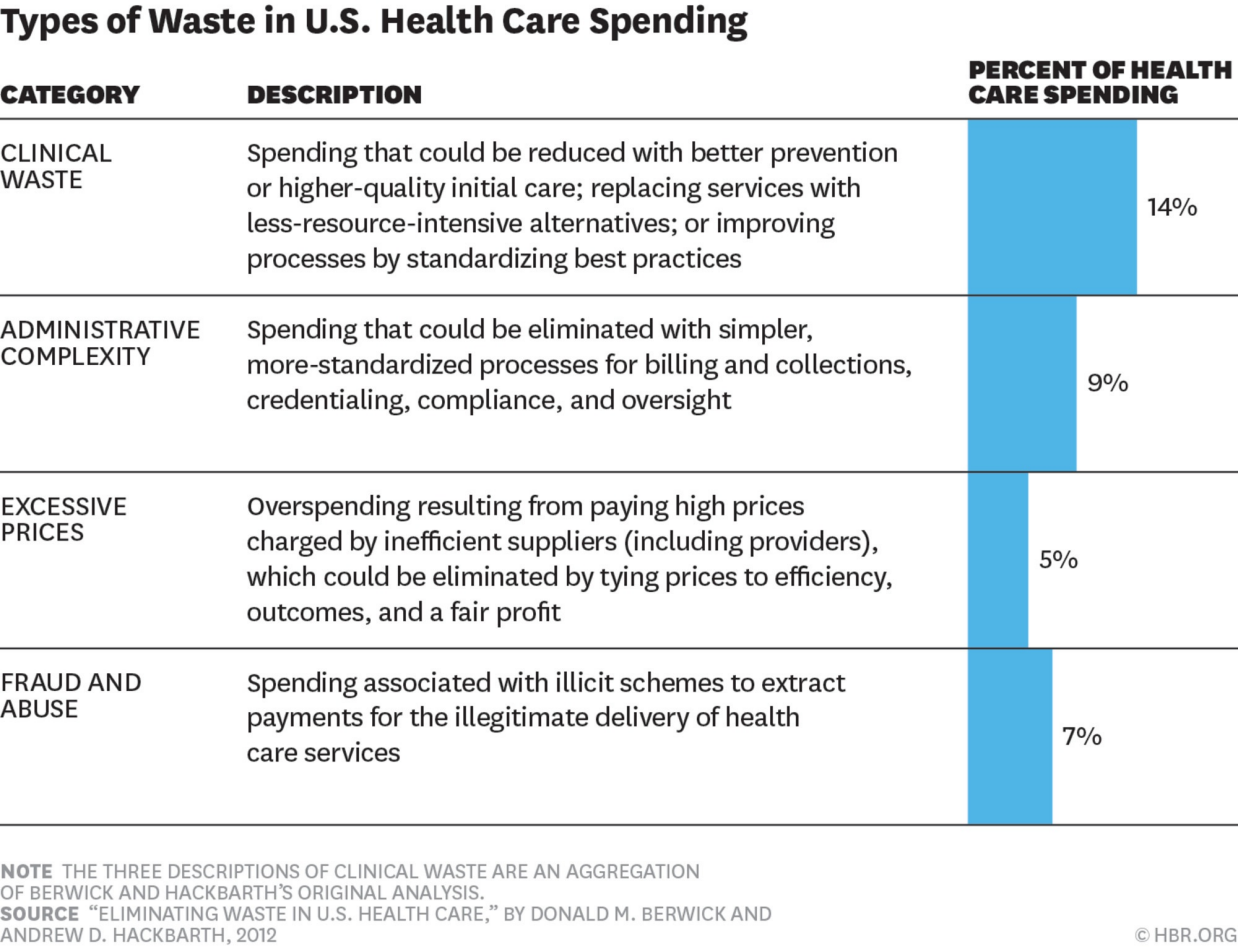
Common Types of Waste in the United States (US) Healthcare System Adopted from Reference #32

Several other factors are contributing to the escalating health care spending growth in the US. A major proportion is attributed to waste in health care. According to a conservative estimate, waste in health care attributes to almost 20% of the overall health care spending. In the Medicare survey by Colla et al., low-value diagnostic services contributed to a majority of the waste in which a significant proportion came from cardiac screening 12.2% ($9.4 million), preoperative cardiac testing in cataract surgery 15.4% ($0.6 million), and preoperative cardiac testing in noncardiac surgery 46.5% ($3.2 million) [31]. Further waste can be categorized into four major groups [32]. Figure 2 describes these groups and their impact on the economy.

Health care policies and legislative changes are supposed to address several of these factors; however, being the front-runner in the health care system, the health care providers can significantly contribute at an individual level by reducing the waste in health care. The introduction of ABIM’s “Choosing Wisely” campaign is an important step in this direction.

Researchers have tried to assess the economic impact of the implications and outcomes of high-value care and the Choosing Wisely guidelines, but several limitations have made it very difficult to precisely measure these impacts. One of the major limitations is to have a practical measuring definition that could be used as inclusion criteria for studies on low-value care [33]. In a study focusing on measuring the low-value care on Medicare beneficiaries, about 26 measures were studied. For each measure, two types of criteria were used. One type of criteria was more sensitive (but less specific), while the other type was more specific (but less sensitive). Using these two types of criteria for each of the 26 services, about 5% of the Medicare beneficiary population was randomly selected and their claims were analyzed using the above-described criteria. The services which were studied were those that were described as low-value services as per the “ABIM Choosing Wisely” initiative, the U.S. Preventive Services Task Force (USPSTF) “D” recommendations, the National Institute for Health and Care Excellence “do not do” recommendations, and the Canadian Agency for Drugs and Technologies in Health Technology assessments or peer-reviewed studies. By using the more sensitive criteria, it was found that about 42% of the beneficiaries received at least one low-value service, while by using the more specific criteria, this figure goes down to 25% of beneficiaries receiving at least one low-value service. This leads to a projected spending of about $8.5 billion or $1.9 billion based on the sensitive and specific criteria that were used, respectively. This comprises about 2.7% and 0.6%, respectively, of the total of Medicare parts A and B annual spending in 2009. Hence, about one-fourth of Medicare beneficiaries received some kind of low-value care. Since this study used Medicare type A and B claims-based measures, there is a high likelihood of under-representation of the actual overall burden of low-value care.

Awareness of the Choosing Wisely campaign

The goal of the Choosing Wisely campaign is to initiate a discussion between the providers and the patients regarding the meaningful and high-value care and avoidance of harm attributed to wasteful care. This can be achieved by increasing the awareness and education of patients through awareness campaigns and educational seminars in community centers, schools, colleges, places of religious gatherings (such as churches), and on mass media, as well as social media (including television, radios, newspapers, Facebook, Twitter, Google, etc.). Similarly, the compliance of providers with these guidelines may be improved by awareness campaigns and educational seminars. Also, training sessions for the providers on high-value care need to be a part of their clinical training, academic curriculum, and board examinations. As the waste in healthcare is so enormous (at least 20% of the total health care expenditure which is about $558 billion), even a slight reduction in this waste would have a significantly beneficial impact on the overall health care system. This exercise would need highly bold, sincere, and honest leadership attributes and a change of culture in our health care system, as well as in our communities. A shift in the culture of the communities in terms of diet and habits is also deemed necessary. Considering the increased prevalence and incidence of obesity and other cardiovascular diseases, we may need to consider a discouraging culture for consumption of tobacco, high fat and high-calorie diets, alcohol, etc.

A significant amount of healthcare waste is also attributed to defensive medicine when a provider pursues an overly zealous investigative workup under the pressure of or to avoid any imminent legal action. We will have to get rid of defensive medicine, and for that to happen, legislative changes would also be required in order to provide some coverage and legal leverage for the healthcare providers.

Learning lessons from the experiences of other nations, such as the National Health Service (NHS) in the United Kingdom, may also be helpful. Training the staff in quality improvement is important [34]. The NHS at some time was facing a similar problem of the increasing costs of healthcare. A series of programs based upon improvement methodologies were introduced, such as the Lean and Six Sigma methodologies, to identify the waste in health care and possible solutions to get rid of such waste [34]. These programs helped their system significantly.

The use of electronic health records and information technology have been very instrumental in decreasing health care costs in the long run. This would avoid repetition of the investigations and would save time and energy consumed in reading and maintenance of paper records. The ACA has already addressed this aspect and offers incentives to facilities equipped with an electronic health record (EHR) system.

Sometimes patients are driven by media advertisements and actively demand inappropriate investigations or treatment. Physicians feel compelled to order unnecessary tests due to several reasons and go in agreement with the patient. The reasons may be patient satisfaction based on financial incentives or practice rankings and reputations. Physicians need to devote more time to such patients for proper discussion and education regarding the appropriateness of the management. However, in the current financial structure, instead of being rewarded, the physician is most likely to lose time, patients, incentives, etc. Issues like these also need to be addressed in the health policy and structural designing [35].

## Conclusions

The CWC has grown significantly beyond what was anticipated at its beginning. The main aim is to avoid unnecessary testing and the constraint of healthcare spending without affecting patient care and adverse outcomes. There are many challenges to face in its adoption and spread, including challenges from the provider, patient, pharmaceutical companies, and insurance. The campaign has been well-recognized internationally. It needs to demonstrate improving outcomes with no hazard to the quality and safety of the patient, which is very important for both providers and patients.   
